# MAINTENANCE OF HEALTH BEHAVIOR CHANGE: RECOMMENDATIONS FOR FUTURE RESEARCH

**DOI:** 10.1093/geroni/igad104.1292

**Published:** 2023-12-21

**Authors:** Jaime Hughes, Aliza Randazzo, Rahma Ajja

**Affiliations:** Wake Forest School of Medicine, Winston-Salem, North Carolina, United States; Wake Forest University School of Medicine, Winston-Salem, North Carolina, United States; Wake Forest University School of Medicine – Winston-Salem, North Carolina, Winston Salem, North Carolina, United States

## Abstract

Modifying poor health behaviors can enhance function, improve quality-of-life, and reduce morbidity and mortality among older adults. However, achieving and sustaining behavior change can be difficult. A growing body of research suggest that initiation and maintenance are unique constructs within health behavior change; each requiring unique skills and each associated with unique outcomes. Although some leading health behavior change models (e.g., Transtheoretical Model of Change) include maintenance as a construct, maintenance is often overlooked in the scientific literature. As part of a NIA Research Centers Collaborative Network (RCCN) pilot award, this project utilized a series of qualitative activities to explore the concept of maintenance and identify future recommendations for advancing the field. Three unique listening sessions were conducted with clinician-researchers (1 group) and community-based service organizations (2 groups). An interdisciplinary expert think tank was then convened to reflect on key findings. All sessions were conducted virtually; transcripts were reviewed using a priori themes with additional themes generated by an open coding approach. Major themes included the following: (1) To date, there is no consensus definition of maintenance nor is there agreement on maintenance across behaviors. (2) Few behavior change models consider how health behaviors must shift to accommodate for age-related changes in cognition and/or physical function. (3) Existing funding mechanisms and timelines may not allow sufficient follow-up periods to examine maintenance over time. (4) Although the terms maintenance and sustainment are used interchangeably, community leaders emphasized the importance of sustaining health promotion programs in order to enable maintenance to occur.

